# New Appliances and Things Medical

**Published:** 1904-04-30

**Authors:** 


### NEW APPLIANCES AND THINGS MEDICAL.

[We shall be glad to receive, at our Office, 28 & 29 Southampton Street Strand, London, W.C., from the manufacturers, specimens of all new preparations and appliances which may be brought out from time to time.]

### THE SPARKLET SYPHON.

(Aerators, Limited, Angel Road, Edmonton.)

There are numerous reasons for the adoption of the sparklet syphon, and we are aware of none against it. The use of the syphon enables the public to secure an aerated table water of greater purity and cleanliness than they are equally certain of obtaining through the medium of syphons charged commercially. It is not necessary to go far to satisfy oneself that the majority of syphons in the market exhibit a marked want of cleanliness. There are, of course, firms who exercise due care, but their products are proportionately expensive, and consequently there is a demand for the cheaper and less cleanly article. The use of the sparklet syphon actually effects an economy. It is readily charged, can be speedily taken to pieces for cleaning, and the metal head is free from anything deleterious under the action of the water. It is protected by a network of wire. Under these circumstances the sparklet syphon ought to become popular in all households. It is very convenient for use in the country, where aerated waters are not readily obtainable and anyhow are cumbersome to store.

